# Comprehensive Analysis of Myoferlin in Human Pancreatic Cancer via Bioinformatics

**DOI:** 10.1155/2021/2602322

**Published:** 2021-12-16

**Authors:** Rou Pi, Yanmei Chen, Yijie Du, Suzhen Dong

**Affiliations:** ^1^Shanghai Engineering Research Centre of Molecular Therapeutics and New Drug Development, School of Chemistry and Molecular Engineering, East China Normal University, Shanghai 200062, China; ^2^Department of Integrative Medicine, Huashan Hospital, Fudan University, Shanghai 200040, China; ^3^Institute of Integrative Medicine, Fudan University, Shanghai 200040, China

## Abstract

Pancreatic cancer is the fourth leading cause of cancer-related death and urgently needs biomarkers for clinical diagnosis and prognosis. It has been reported that myoferlin (MYOF) is implicated in the regulation of proliferation, invasion, and migration of tumor cells in many cancers including pancreatic cancer. To confirm the prognostic value of MYOF in pancreatic cancer, a comprehensive cancer versus healthy people analysis was conducted using public data. MYOF mRNA expression levels were compared in many kinds of cancers including pancreatic cancer via the Oncomine and Gene Expression Profiling Interactive Analysis (GEPIA) databases. The results have shown that MYOF mRNA expression levels were upregulated in most types of cancers, especially in pancreatic cancer, compared with healthy people's tissues. Data from the Cancer Cell Line Encyclopedia (CCLE) and European Bioinformatics Institute (EMBL-EML) database also revealed that MYOF mRNA is highly expressed in most cancer cells, particularly in pancreatic cancer cell lines. Furthermore, the prognostic value of MYOF was evaluated using GEPIA and Long-term Outcome and Gene Expression Profiling Database of pan-cancers (LOGpc) database. Higher expression of MYOF was associated with poorer overall survival, especially in the lower stage and lower grade. Coexpressed genes, possible regulators, and the correlation between MYOF expressions were analyzed via the GEPIA and LinkedOmics database. Nineteen coexpressed genes were identified, and most of these genes were related to cancer. The Tumor Immune Estimation Resource (TIMER) database was used to analyze the correlation between MYOF and immune response. Notably, we found that MYOF might have a potential novel immune regulatory role in tumor immunity. These results support that MYOF is a candidate prognostic biomarker for pancreatic cancer, which calls for further genomics research of pancreatic cancer and deeply functional studies on MYOF.

## 1. Introduction

Myoferlin (MYOF), a member of the ferlin family, is a multiple-C2-domain-containing type II transmembrane protein. It is involved in many important cellular processes, such as receptor internalization and recycling, endocytosis, exocytosis, and the maintenance of intercellular membrane structures [1]. MYOF dysfunction is associated with human muscle atrophy [2].

Besides, increasing studies have shown that MYOF might be an oncogenic protein. MYOF dysfunction has been found in breast cancer, pancreatic adenocarcinoma, hepatocellular carcinoma, melanoma, oropharyngeal squamous cell carcinoma, head and neck squamous cell carcinoma, clear cell renal cell carcinoma, endometrioid carcinoma, and so on [3]. It has been demonstrated that MYOF has an important role in the regulation of proliferation, invasion, and migration of tumor cells via mechanisms including promotion of angiogenesis, vasculogenic mimicry, energy metabolism reprogramming, epithelial-mesenchymal transition, and modulation of exosomes [3]. Hence, MYOF might be a promising target for clinical diagnosis and treatment of malignant tumors.

Pancreatic cancer is the fourth leading cause of cancer-related deaths in 2017, and it is estimated that about 57,600 new cases and 47,050 cases of pancreatic cancer-related deaths occur in 2020 in the United States [4]. Pancreatic cancer often does not show symptoms until it reaches the advanced stages of the disease, which makes pancreatic cancer patients have a 5-year survival rate of only 8%. Therefore, a comprehensive analysis of pancreatic cancer to identify biomarkers for clinical diagnosis and prognosis is urgently needed.

MYOF has been identified to be associated with pancreatic cancer diagnosis and treatment. MYOF has been shown to be overexpressed in pancreatic tumors based on whole-genome gene expression profile analysis [5–7] and proteomics results [8–10]. MYOF is implicated in the regulation of vascular endothelial growth factor A (VEGFA) secretion and has an impact on tumor-associated angiogenesis in human pancreatic cancer [11]. Besides, Rademaker et al. found that MYOF is involved in the regulation of tumor aggressiveness by induction of energy metabolism reprograming in lipogenic pancreatic cancer cell lines [12]. Furthermore, compounds targeting MYOF have been shown to inhibit pancreatic cancer metastasis by reversing the epithelial mesenchymal transition, suppressing the secretion of matrix metalloproteinase and blocking the receptor tyrosine kinases [13].

The dysregulated expression level of MYOF and its relationship with clinicopathological features and prognosis have been partly reported in human pancreatic cancer [11, 12]. To confirm that MYOF can be a biomarker for pancreatic cancer diagnosis and prognosis, a comprehensive bioinformatics analysis should be done. Here, we are trying to perform a detailed analysis on the expression, prognosis, and coexpressed protein network and immune analysis of MYOF in patients with pancreatic cancer to determine its expression patterns, potential functions, and distinct prognostic values in pancreatic cancer based on data from public databases.

## 2. Materials and Methods

### 2.1. Oncomine Analysis

Oncomine (https://www.oncomine.org/) is an online cancer-related microarray database and data-mining platform. Gene expression array datasets in this database (version 4.5) were used to analyze MYOF mRNA expression levels in different cancers. MYOF mRNA expression levels in clinical cancer specimens were compared with those in healthy controls using Student's *t*-test. The threshold was determined according to the following values: *P* value of 0.01 and fold change of 2.

### 2.2. GEPIA Dataset

GEPIA (Gene Expression Profiling Interactive Analysis) is a web server including mRNA expression data of 9,736 tumors and 8,587 healthy samples from the Genotype Tissue Expression (GTEx) and The Cancer Genome Atlas (TCGA) [14] projects (http://GEPIA.cancer-pku.cn/). MYOF mRNA expression, prognosis, and coexpression were analyzed in the GEPIA database. And a correlation analysis between MYOF expression and a signature of coexpressed genes was also performed using GEPIA2.

### 2.3. The Human Protein Atlas Database

MYOF expressed in human healthy people's tissues and tumor tissues was validated via the Human Protein Atlas (HPA, https://www.proteinatlas.org/, version 19.3). The Human Protein Atlas is a Swedish-based project initiated in 2003 with the aim of mapping all human proteins in cells, tissues, and organs using the integration of various omics technologies, including antibody-based imaging, mass spectrometry-based proteomics, transcriptomics, and systems biology. The database provides the protein expression information for 44 major human tissues and some cancer tissues using immunohistochemistry methods [15]. The HPA014245 dataset was used in the present study. Statistical analysis was performed using ImageJ and GraphPad Prism 8, and the *P* value was determined using the *t*-test. Statistical significance was accepted as *P* < 0.05.

### 2.4. CCLE Dataset

The CCLE (https://www.broadinstitute.org/ccle) is a project initiated by the Broad Institute to conduct a detailed genetic and pharmacologic characterization of a large panel of human cancer models [16]. The CCLE provides public access to genomic data, expression analysis, and visualization for 1457 cell lines. The MYOF expression in cancer cell lines was analyzed using the CCLE dataset.

### 2.5. EMBL-EBI Dataset

EMBL-EBI (https://www.ebi.ac.uk) has provided free and open access to the world's most comprehensive range of molecular databases and an extensive user training program [17]. MYOF mRNA expression in pancreatic cancer cell lines is validated by the EMBL-EBI dataset.

### 2.6. LinkedOmics Database Analysis

The LinkedOmics database (http://www.linkedomics.org/login.php) is an online platform for analyzing 32 TCGA cancer-associated datasets [18]. MYOF coexpression was analyzed statistically using Pearson's correlation coefficient, and the results were presented in volcano plot heat maps. The analysis of Gene Ontology biological process (GO_BP), KEGG pathways, and possible kinase, miRNA, and transcription factor regulators of MYOF enrichment was performed by gene set enrichment analysis (GSEA) using the function module of LinkedOmics. The rank criterion was FDR < 0.05, and 500 simulations were used.

### 2.7. TIMER Database Analysis

TIMER is a comprehensive web server for systematic analysis of immune infiltrates of six kinds of immune cells (B cells, CD4^+^ T cells, CD8^+^ T cells, neutrophils, macrophages, and dendritic cells) across diverse cancer types from TCGA (https://cistrome.shinyapps.io/TIMER/) [19]. TIMER used a deconvolution algorithm to estimate the abundance of tumor-infiltrating immune cells (TIICs) based on gene expression profiles. We used this database to analyze the correlation of MYOF expression with the abundance of TIICs and tumor purity (*P* < 0.05).

## 3. Results

### 3.1. Transcriptional Levels of MYOF in Patients with Pancreatic Cancer

**Figure 1:**
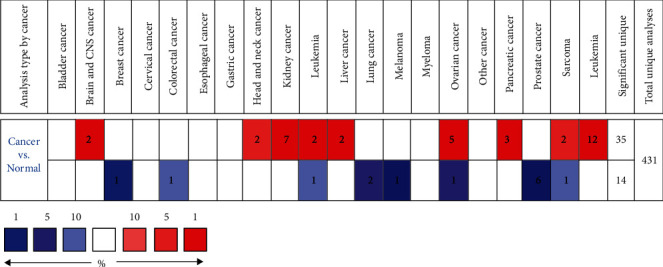
The transcription levels of MYOF in different types of cancers (Oncomine). The figures stand for the number of studies that meet the threshold of our analysis.

The Oncomine database was used to compare the transcription levels of MYOF in cancer and healthy people's samples (Figure 1). The results showed that the expression levels of MYOF were higher in tumor samples than in healthy samples in most cancers. Notably, MYOF mRNA expression level was significantly upregulated in 6 datasets of pancreatic cancer patients (Table 1). In particular in Segara pancreas' dataset, MYOF was highly expressed compared to that of the healthy samples with a fold change of 7.026. In Badea pancreas' and Iacobuzio-Donahue pancreas' dataset, MYOF was also overexpressed with a fold change of 5.159 and 5.898, respectively. In the other pancreas' dataset, the fold change is around 2.

**Figure 2:**
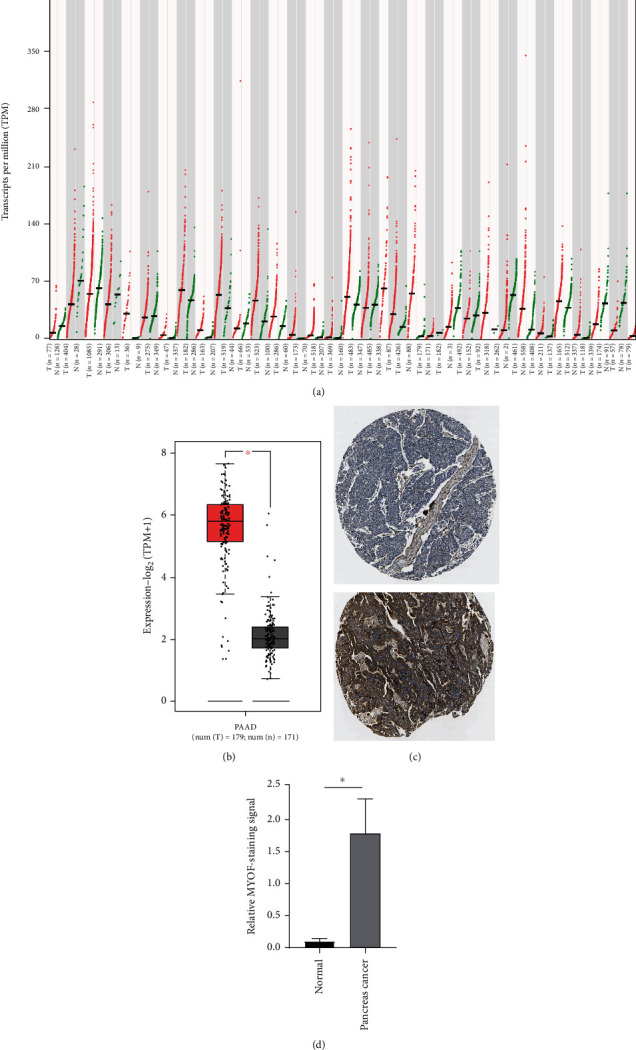
MYOF expression is upregulated in pancreatic cancer patients (GEPIA). (a) The expression of MYOF in pan-cancer. PAAD: pancreatic cancer. (b) MYOF mRNA level is much higher in pancreatic cancer patients than in healthy people (*P* < 0.05). (c) Representative images of MYOF immunostaining in healthy tissue and pancreatic cancer tissue (HPA014245). Up: normal pancreas; down: pancreatic cancer tissue. (d) Statistical analysis of MYOF immunostaining signals in healthy and pancreatic cancer tissues (*P* < 0.05).

MYOF mRNA expression levels between pancreatic cancer and healthy samples were compared again using the Gene Expression Profiling Interactive Analysis (GEPIA, http://gepia.cancer-pku.cn/detail.php). The results also indicated that MYOF mRNA levels were upregulated in pancreatic patients (Figures 2(a) and 2(b)). Notably, MYOF was highly expressed in every stage, and higher expression was found at advanced stages (Figure S1). MYOF expression at protein levels was further analyzed using the Human Protein Atlas database (HPA, https://www.proteinatlas.org/). Similar results were observed. MYOF protein levels in pancreatic cancer tissues were significantly overexpressed. Therefore, the expression of MYOF in pancreatic cancer increased at both mRNA and protein levels.

### 3.2. MYOF Expression in Pancreatic Cancer Cell Lines

**Figure 3:**
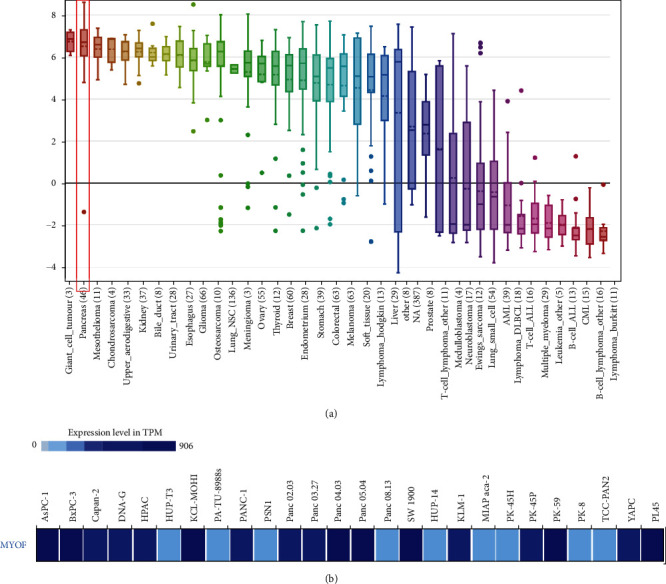
The expression of MYOF mRNAs in the cell lines of pancreatic cancer (CCLE and EMBL-EBI): (a) the expression of MYOF in pancreatic cancer and other cancer cell lines analyzed by CCLE; (b) the expression of MYOF in pancreatic cancer cell lines analyzed by EMBL-EBI.

Next, we consulted the Cancer Cell Line Encyclopedia (CCLE, https://www.broadinstitute.org/ccle) to see whether MYOF is highly expressed in pancreatic cancer cells. We found that most pancreatic cancer cells expressed MYOF highly (Figure 3(a)). The expression of MYOF in pancreatic cancer cell lines was also analyzed in the European Bioinformatics Institute (EMBL-EBI) bioinformatics website (https://www.ebi.ac.uk/gxa/home). The results indicated that MYOF was overexpressed in most cell lines of pancreatic cancer (Figure 3(b)).

### 3.3. The Prognostic Value of MYOF in Pancreatic Cancer

**Figure 4:**
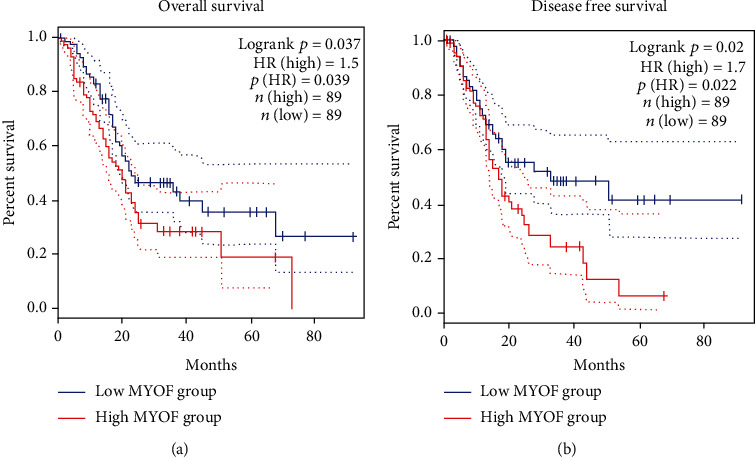
The prognostic value of MYOF mRNA level in pancreatic cancer patients (GEPIA): (a) the OS of MYOF in pancreatic cancer patients (*P* = 0.039); (b) the DFS of MYOF in pancreatic cancer patients (*P* = 0.022).

**Figure 5:**
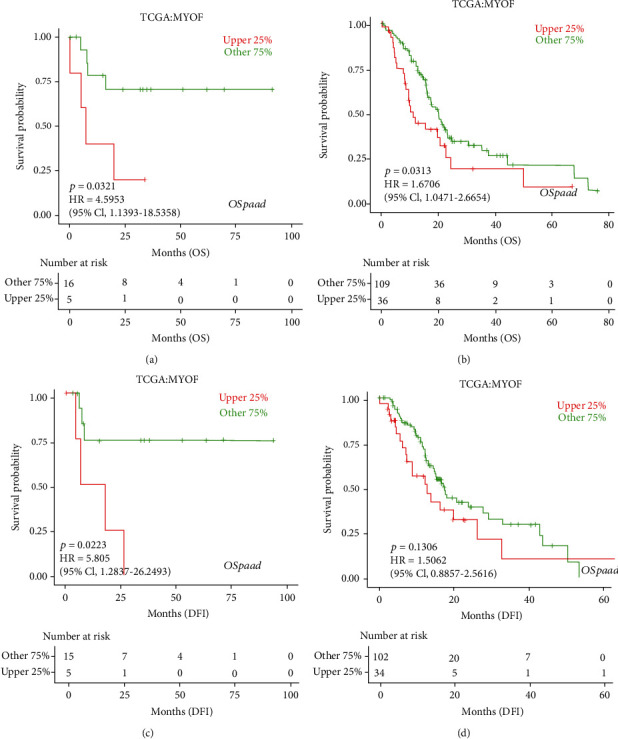
The prognostic value of MYOF mRNA level in pancreatic cancer patients at different stages (LOGpc): (a) the OS of MYOF in stage I pancreatic cancer patients (*P* = 0.0321); (b) the OS of MYOF in stage II pancreatic cancer patients (*P* = 0.0313); (c) the DFI of MYOF in stage I pancreatic cancer patients (*P* = 0.0223); (d) the DFI of MYOF in stage II pancreatic cancer patients (*P* = 0.136).

**Figure 6:**
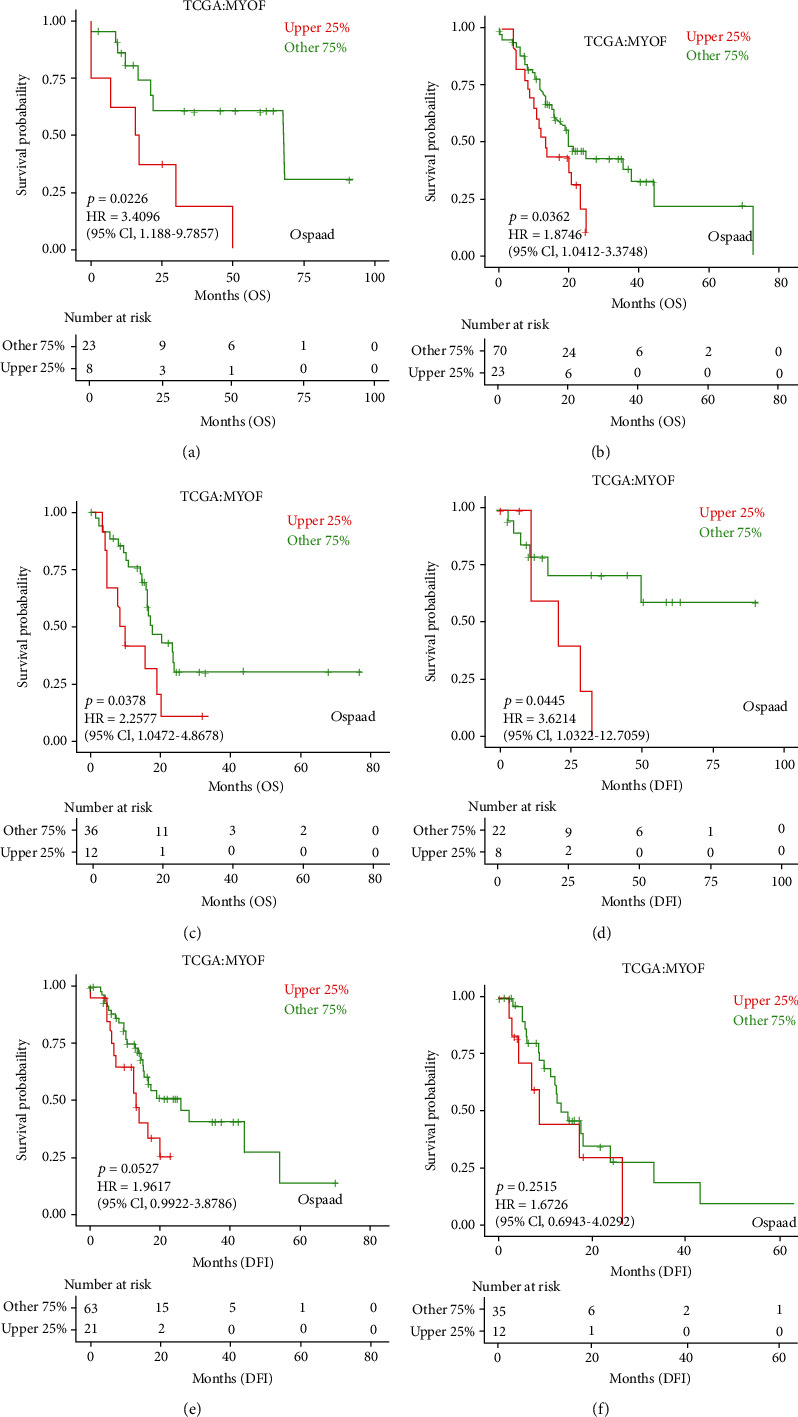
The prognostic value of MYOF mRNA level in different-grade pancreatic cancer patients (LOGpc): (a) the OS of MYOF in grade 1 pancreatic cancer patients (*P* = 0.0226); (b) the OS of MYOF in grade 2 pancreatic cancer patients (*P* = 0.0362); (c) the OS of MYOF in grade 3 pancreatic cancer patients (*P* = 0.0378); (d) the DFI of MYOF in grade 1 pancreatic cancer patients (*P* = 0.045); (e) the DFI of MYOF in grade 2 pancreatic cancer patients (*P* = 0.0527); (f) the DFI of MYOF in grade 3 pancreatic cancer patients (*P* = 0.2515).

Then, we performed prognosis analysis for MYOF in pancreatic cancer using the GEPIA database. Increased MYOF is associated with poor overall survival (OS) and poor disease-free survival (DFS) in pancreatic cancer (Figures 4(a) and 4(b)). Moreover, further investigation on the OS and disease-free interval (DFI) in different stages, grades, and genders was done by using the LOGpc database (http://bioinfo.henu.edu.cn/DatabaseList.jsp). The results have shown that increased MYOF level may be associated with poor OS at stages I and II and poor DFI in stage I (Figures 5(a)–5(d)). However, no such relationship can be observed in stages III and IV (Figure S2A-B), possibly because samples of pancreatic cancer at advanced stages were limited. As for different grades of pancreatic cancer, increased MYOF expression was significantly associated with poor OS in every grade but poor DFI only in grade 1 in males (Figures 6(a)–6(f)). However, there were no significant differences in OS or DFI between the high-expressed and low-expressed female patients indicated by the LOGpc database (Figure S3A-D).

### 3.4. MYOF Coexpression Networks in Pancreatic Cancer

**Figure 7:**
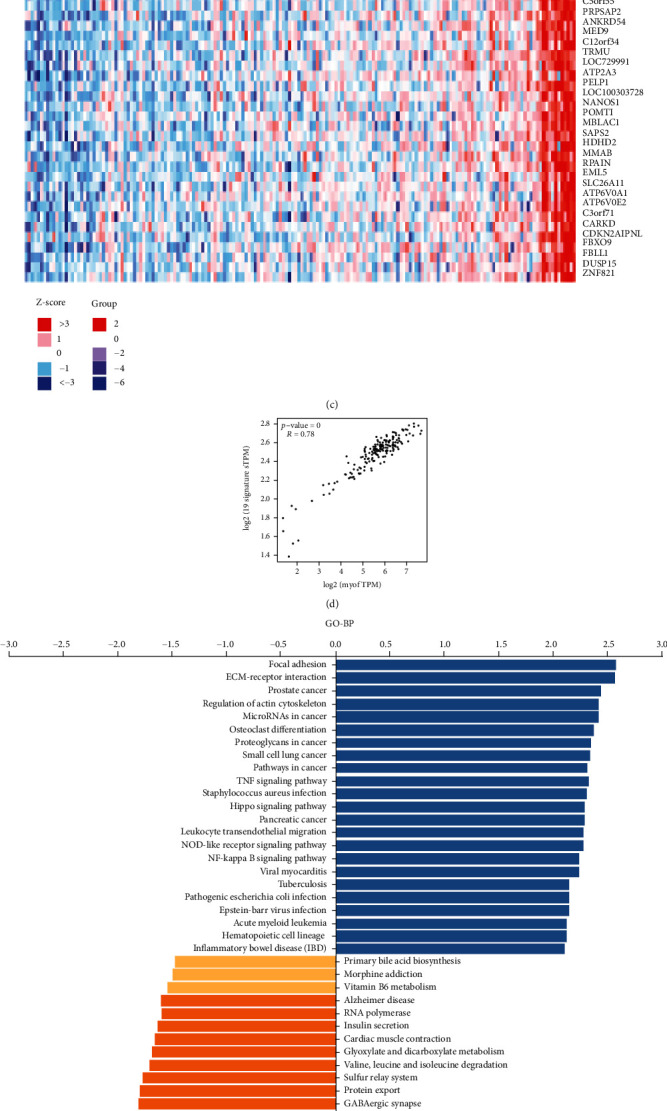
MYOF coexpression genes in pancreatic cancer (LinkedOmics): (a) the global MYOF highly correlated genes identified by the Pearson test; (b, c) Heat maps have shown that the top 50 genes positively and negatively correlated with MYOF in pancreatic cancer. Red indicates positively correlated genes, and blue indicates negatively correlated genes; (d) the association between MYOF expression and coexpressed gene signature was validated in the GEPIA database; (e, f) Significantly enriched GO annotations and KEGG pathways of MYOF coexpressed genes.

To gain the insight of MYOF biological meaning in pancreatic cancer, LinkedOmics and GEPIA databases were used to elucidate the coexpressed proteins of MYOF in the PAAD (pancreatic cancer) cohort. At first, we used LinkedOmics to find genes that have positive or negative correlations with MYOF. The results are shown in Figure 7(a). The top 50 significant genes positively and negatively correlated with MYOF were shown in the heat map (Figures 7(b) and 7(c); the details are shown in Table S1). Then, the top one hundred genes similarly expressed with MYOF in pancreatic cancer were identified using GEPIA2. Nineteen genes (FGD6, MET, YAP1, PLS3, RUNX1, PTPN12, ARHGAP42, EPS8, ITPRIPL2, VCL, AHNAK, TMOD3, RALB, TPM4, AFAP1, RYK, FRMD6, PTPN14, and REEP3) were both found in the lists of the most correlated genes identified by LinkedOmics and GEPIA2. A strong correlation was found between MYOF and the 19-gene signature analyzed using GEPIA2 (*P* = 0, *R* = 0.78, Figure 7(d)).

Significant Gene Ontology (GO) term annotations were performed by gene set enrichment analysis (GSEA). The results have shown that MYOF coexpressed genes participate primarily in extracellular structure organization, cell-substrate adhesion, cell adhesion mediated by integrin, cell junction organization, positive regulation of cell adhesion, angiogenesis, leukocyte cell-cell adhesion, muscle cell migration, epithelial cell proliferation, and many other processes (FDR = 0). Meanwhile, mitochondrial respiratory chain complex assembly, NADH dehydrogenase complex assembly, and energy processes (FDR = 0) were inhibited (Figure 7(e)). Kyoto Encyclopedia of Genes and Genomes (KEGG) pathway analysis indicated significant enrichment in the ECM-receptor interaction, focal adhesion, regulation of actin cytoskeleton, proteoglycans in cancer, microRNAs in cancer, etc. (Figure 7(f)). These results suggested an extensive impact of MYOF on the global transcriptome in pancreatic cancer.

### 3.5. Regulator of the MYOF Network in Pancreatic Cancer

To further explore the possible regulators of MYOF in pancreatic cancer, we analyzed the kinase, miRNA, and transcription factor [24] enrichment of MYOF coexpressed genes using the LinkedOmics database (Figure S4A-C).

CDK1, MAPK3, ABL1, EGFR, and MAPK1 were the top five significant kinases enriched in MYOF coexpressed genes. All these kinase genes were involved in the regulation of cell proliferation and cell cycle. Besides, these kinases were significantly overexpressed in many tumor tissues. In addition, CDK1 and EGFR were significantly associated with the OS of pancreatic cancer (Figure S5A-B). The top five miRNAs were miR-26A, miR-26B, miR-188, miR-374, miR-189, and miR-145. And the top five transcription factors [24] were V$IRF_Q6, V$NRSF_01, V$SRF_C, V$CEBPB_02, and V$ICSBP_Q6 (Table 2).

### 3.6. MYOF Is Correlated with Tumor Purity and Immune Infiltration Level in Pancreatic Cancer and Is Associated with Immune Signatures

**Figure 8:**
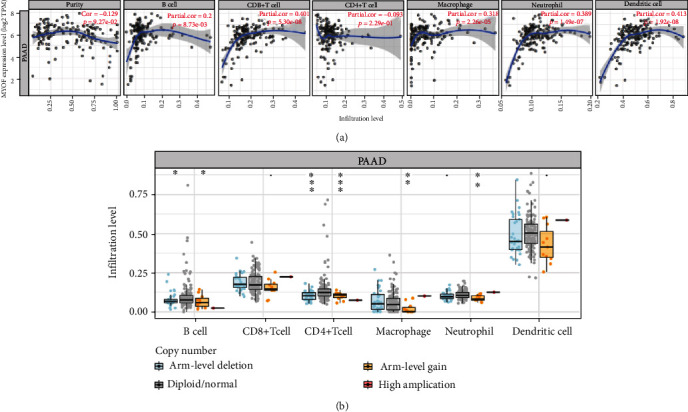
Correlation of MYOF expression with immune infiltration level in pancreatic cancer (TIMER): (a) MYOF expression has significant positive correlations with infiltrating levels of B cells, CD8^+^ T cells, macrophages, neutrophils, and dendritic cells in pancreatic cancer; (b) MYOF copy number variation is associated with the infiltrating levels of B cells, CD4^+^ T cells, macrophages, and neutrophils in pancreatic cancer.

Dysferlin, a protein with similar structure and function to MYOF, is associated with immune response [25]. Meanwhile, inflammation has emerged to be a key mediator of pancreatic cancer development [26]. Therefore, we speculated that the role of MYOF in pancreatic cancer might be also related to immunity and inflammation. The TIMER (https://cistrome.shinyapps.io/timer/) database was used to explore this issue. Although the correlation between MYOF expression and tumor purity was not very significant (*R* = 0.129, *P* = 9.27*e* − 02), MYOF was correlated with some dominant immune cell infiltration levels, such as B cells, CD8^+^ T cells, macrophages, neutrophils, and dendritic cells (Figure 8(a)). Besides, MYOF copy number variation also had a significant correlation with B cells, CD4^+^ T cells, macrophages, neutrophils, and dendritic Cells (Figure 8(b)).

Finally, to enhance the understanding of MYOF crosstalk with immune-related genes, we further analyzed the correlations between MYOF expression and various immune signatures, which included immune marker genes of 28 tumor-infiltrating lymphocytes (TILs), immune stimulatory or inhibitory genes (including immune checkpoint gene sets), cancer-related antigen genes, cytokine-related genes, and major histocompatibility complex (MHC) genes (Table 3).

## 4. Discussion

MYOF dysfunction has been demonstrated to be related to proliferation, aggressiveness, and angiogenesis of many cancers. Previous studies have shown that MYOF might be a candidate biomarker for the diagnosis and prognosis of pancreatic cancer [5–10]. Here, we systematically analyzed the expression, prognosis, and coexpressed genes of MYOF in pancreatic cancer patients using public data. Besides, the correlation of MYOF expression and immune infiltrates was also studied. The results display that MYOF is abnormally highly expressed in pancreatic cancer and the upregulation is associated with poor prognosis. Meanwhile, 19 genes have been found to be coexpressed with MYOF, many of which were cancer-related genes. In addition, some well-known oncogenic kinases and miRNAs were possible regulators of MYOF. And a significant correlation was found between MYOF expression and the infiltrates of some immune cells. These results further supported that MYOF could be an excellent biomarker for pancreatic cancer. We hope that our findings will be helpful for improving therapy design and enhancing the accuracy of prognosis for patients with pancreatic cancer.

Our study shows that MYOF is more highly expressed in both pancreatic cancer patients and pancreatic cancer cell lines than in healthy tissues and cells. We also proved that MYOF expression level was also associated with the stage of pancreatic cancer, with higher expression at advanced stages (Figure S1). Besides, datasets from GEPIA and LOGpc reveal that high MYOF expressions in pancreatic cancer patients are significantly associated with unfavorable prognosis. Hence, our results indicate that increased expression of MYOF occurs in many cases of pancreatic cancer and deserves further clinical validation as a candidate biomarker for diagnosis and prognosis.

To further confirm the prognostic value of MYOF, coexpressed genes of MYOF were comprehensively analyzed using both GEPIA and LinkedOmics databases. Nineteen coexpressed genes were identified by both databases. Nine of these genes (FGD6, PLS3, EPS8, VCL, AHNAK, TMOD3, TPM4, AFAP1, and PTPN14) encode actin-binding proteins or are associated with cytoskeleton. GO annotation and KEGG pathway analysis both showed that genes related to cell adhesion and cytoskeleton were enriched in coexpressed genes of MYOF. Notably, nine genes (MET, YAP1, RUNX1, PTPN12, EPS8, AHNAK, RALB, AFAP1, and PTPN14) have been demonstrated to play important roles in the processes of cancer, cell cycle regulation, and migration, or invasion of cancer cells. All these genes are upregulated in pancreatic cancer, and seven of them are significantly associated with unfavorable prognosis (MET, YAP1, PTPN14, EPS8, AHNAK, RALB, and AFAP1; Figure S6A-G). MET has been identified as a diagnostic and prognostic marker for pancreatic cancer [27, 28]. YAP-1 has also been demonstrated to be involved in tumor initiation and progression in pancreatic cancer [29, 30]. PTPN14 encodes an inhibitor of the Yap oncoprotein and is involved in pancreatic cancer suppression [31]. Eps8 is upregulated in pancreatic cancer and correlates with migration ability and tumor progression [32]. It is also identified as a metastatic biomarker for pancreatic cancer [33]. AHNAK, a MYOF-interacting protein [34], mediates epithelial-mesenchymal transition, and its overexpression is correlated with the unfavorable outcome of pancreatic ductal adenocarcinoma [35]. RALB has been proven to play an important role in pancreatic ductal adenocarcinoma (PDAC) tumorigenesis and invasive and metastatic growth [36]. AFAP1 is an actin-binding protein that might be critical for tumorigenic growth [37]. Hence, MYOF coexpresses with many cancer-related genes in pancreatic cancer, which also support its prognostic value.

MYOF expression increases in pancreatic cancer. We wonder which regulator might participate in the regulation of this process. Hence, the possible regulator of MYOF was analyzed using the LinkedOmics database. We found that the top 5 significantly enriched kinases are CDK1, MAPK3, ABL1, MAPK1, and EGFR. All these kinases are known to be oncogenic and dysfunctional in cancer. EGFR has been identified to be regulated by MYOF in breast cancer [38]. Whether MYOF can be affected by EGFR and other kinases needs further investigation. Among the significantly enriched miRNAs, miR-374 can be predicted to target MYOF. However, whether MYOF is a target of miR-374 should be validated experimentally. Although the mined information needs to be confirmed, it shows the direction for further experiments.

Immune response is implicated in the tumorigenesis of pancreatic cancer [26] and dysferlinopathy mediated by dysferlin [25], a similar protein of MYOF. Here, we found that MYOF may be associated with immune signatures and MYOF CNV affects the infiltrating levels of most immune cells. It indicated that MYOF may participate in the immune process. Enhanced membrane repair involving myoferlin, dysferlin, and annexins has been found to be accompanied by a robust inflammatory response in the progressive neuromuscular disorder dystrophinopathy [39]. Whether MYOF dysfunction in cancer is related to the immune response has not been reported until now. Here, we found that there is a possible link between them in pancreatic cancer using public data. This issue needs more researches to confirm.

Genomic instability is a characteristic of tumors, and initiation of many cancers is associated with gene mutation. Hence, we used the cBioPortal tool to analyze the types and frequency of MYOF alterations in pancreatic cancer. The results revealed that the MYOF gene seldom mutated in pancreatic cancer (1%, Figure S7A). Meanwhile, no significant difference can be observed in the survival time between the altered group and unaltered group (Figure S7B-F), possibly because of the limited number of pancreatic cancer cases with altered MYOF.

In summary, this study provides many evidences at different levels for the key role of MYOF in pancreatic cancer and its potential as a prognostic biomarker in it. We found that it is upregulated and an unfavorable prognostic factor. Notably, we found that MYOF might have a potential novel immune regulatory role in tumor immunity. These results call for further genomics research of pancreatic cancer and deeply functional studies on MYOF.

## Figures and Tables

**Table 1:** The significant changes of MYOF expression in transcription level in different studies of pancreatic ductal adenocarcinoma (Oncomine database).

Studies of pancreatic cancer versus healthy tissues	Fold change	*P* value	*t* test	References
Badea et al.	5.159	1.67*E* − 15	11.253	[7]
Segara et al.	7.026	2.49*E* − 6	6.931	[20]
Pei et al.	2.980	1.70*E* − 5	5.206	[21]
Iacobuzio-Donahue et al.	5.898	2.80*E* − 4	7.102	[5]
Grutzmann et al.	2.647	3.00*E* − 03	3.039	[22]
Ishikawa et al.	1.931	1.00*E* − 02	2.418	[23]

**Table 2:** The kinase, miRNA, and transcription factor enrichment of MYOF coexpressed genes using the LinkedOmics database.

Enriched category	Gene set	Size	Leading edge number	*P* value	FDR
Kinase target	Kinase_CDK1	258	89	0.00*E* + 00	0.00*E* + 00
	Kinase_MAPK3	171	64	0.00*E* + 00	0.00*E* + 00
	Kinase_ABL1	83	31	0.00*E* + 00	0.00*E* + 00
	Kinase_EGFR	46	17	0.00*E* + 00	0.00*E* + 00
	Kinase_MAPK1	197	78	0.00*E* + 00	1.12*E* − 03
miRNA target	MIR-26A, MIR-26B	285	104	0.00*E* + 00	2.35*E* − 03
	MIR-188	70	32	0.00*E* + 00	4.71*E* − 03
	MIR-374	267	91	0.00*E* + 00	5.04*E* − 03
	MIR-189	27	10	2.79*E* − 03	5.10*E* − 03
	MIR-145	215	67	0.00*E* + 00	5.49*E* − 03
Transcription factor	V$IRF_Q6	229	94	0.00*E* + 00	0.00*E* + 00
	V$NRSF_01	93	42	0.00*E* + 00	0.00*E* + 00
	V$SRF_C	201	82	0.00*E* + 00	3.46*E* − 04
	V$CEBPB_02	60	31	0.00*E* + 00	4.15*E* − 04
	V$ICSBP_Q6	231	81	0.00*E* + 00	5.19*E* − 04

**Table 3:** Correlations between MYOF expression and markers of activated T cells (TIMER).

	None		Purity	
	Cor	*P*	Cor	*P*
CD8^+^				
ADRM1	1.9*E* − 01	1.2*E* − 02	2.2*E* − 01	4.2*E* − 03
AHSA1	3.4*E* − 01	3.3*E* − 06	3.9*E* − 01	1.0*E* − 07
C1GALT1C1	3.9*E* − 01	1.1*E* − 07	4.3*E* − 01	3.5*E* − 09
CD69	1.9*E* − 01	1.3*E* − 02	1.7*E* − 01	2.7*E* − 02
L2RB	2.1*E* − 01	5.7*E* − 03	1.7*E* − 01	2.3*E* − 02
CETN3	3.0*E* − 01	5.6*E* − 05	3.6*E* − 01	1.6*E* − 06
CSE1L	4.3*E* − 01	2.6*E* − 09	4.8*E* − 01	4.2*E* − 11
GEMIN6	3.1*E* − 01	3.4*E* − 05	2.7*E* − 01	4.2*E* − 04
MPZL1	5.0*E* − 01	0.0*E* + 00	4.6*E* − 01	2.9*E* − 10
TIMM13	−1.9*E* − 01	1.3*E* − 02	−1.6*E* − 01	4.3*E* − 02
MPZL1	5.0*E* − 01	0.0*E* + 00	4.6*E* − 01	2.9*E* − 10
PIK3IP1	2.5*E* − 01	7.9*E* − 04	2.5*E* − 01	9.1*E* − 04
CD4^+^				
AIM2	1.9*E* − 01	1.2*E* − 02	1.6*E* − 01	3.9*E* − 02
BIRC3	3.5*E* − 01	1.8*E* − 06	3.3*E* − 01	1.2*E* − 05
BRIP1	4.7*E* − 01	2.6*E* − 11	4.7*E* − 01	9.1*E* − 11
CCL20	2.1*E* − 01	4.4*E* − 03	1.6*E* − 01	3.4*E* − 02
ESCO2	4.1*E* − 01	2.2*E* − 08	3.8*E* − 01	2.3*E* − 07
ETS1	4.3*E* − 01	2.9*E* − 09	4.3*E* − 01	4.2*E* − 09
CCNB1	4.7*E* − 01	2.3*E* − 11	4.8*E* − 01	2.7*E* − 11
EXO1	4.0*E* − 01	4.2*E* − 08	3.8*E* − 01	2.1*E* − 07
IARS	3.8*E* − 01	2.3*E* − 07	4.4*E* − 01	2.1*E* − 09
KIF11	4.7*E* − 01	6.3*E* − 11	4.6*E* − 01	2.6*E* − 10
KNTC1	4.1*E* − 01	1.3*E* − 08	4.3*E* − 01	3.5*E* − 09
NUF2	3.6*E* − 01	7.2*E* − 07	3.5*E* − 01	3.3*E* − 06
PRC1	4.8*E* − 01	4.6*E* − 12	4.7*E* − 01	1.5*E* − 10
RGS1	2.9*E* − 01	1.1*E* − 04	2.6*E* − 01	5.3*E* − 04
RTKN2	4.8*E* − 01	1.3*E* − 11	4.6*E* − 01	1.5*E* − 10
SAMSN1	3.0*E* − 01	5.3*E* − 05	2.7*E* − 01	3.4*E* − 04

## Data Availability

All data generated or analyzed during this study are included in this published article or supplementary materials.

## Supplementary Materials

Supplementary Materials Supplementary Figure 1: the expression of MYOF in pancreas cancer in each stage. Supplementary Figure 2: the prognostic value of mRNA level of MYOF in different-stage pancreas cancer patients (LOGpc). Supplementary Figure 3: the prognostic value of mRNA level of MYOF in different-gender pancreas cancer patients (LOGpc). Supplementary Figure 4: kinase, miRNA, and transcription factor (TF) enrichment of MYOF coexpressed genes. Supplementary Figure 5: the prognostic value of mRNA level of CDK1 and EGFR in pancreas cancer patients (GEPIA). Supplementary Figure 6: the prognostic value of mRNA level of MYOF coexpressed genes in pancreas cancer patients (GEPIA). Supplementary Figure 7: the alteration of MYOF in pancreas cancer (cBioPortal). Supplementary Table S1: detail of MYOF coexpression genes (TIMER).
