# Supplementary material for: Comprehensive Analysis of Myoferlin in Human Pancreatic Cancer via Bioinformatics
Source: Biomed Res Int. 2021 Dec 16;2021:2602322. doi: 10.1155/2021/2602322 (PMC8702316; doi:10.1155/2021/2602322)
Supplement: Supplementary Materials — Supplementary Figure 1: the expression of MYOF in pancreas cancer in each stage. Supplementary Figure 2: the prognostic value of mRNA level of MYOF in different-stage pancreas cancer patients (LOGpc). Supplementary Figure 3: the prognostic value of mRNA level of MYOF in different-gender pancreas cancer patients (LOGpc). Supplementary Figure 4: kinase, miRNA, and transcription factor (TF) enrichment of MYOF coexpressed genes. Supplementary Figure 5: the prognostic value of mRNA level of CDK1 and EGFR in pancreas cancer patients (GEPIA). Supplementary Figure 6: the prognostic value of mRNA level of MYOF coexpressed genes in pancreas cancer patients (GEPIA). Supplementary Figure 7: the alteration of MYOF in pancreas cancer (cBioPortal). Supplementary Table S1: detail of MYOF coexpression genes (TIMER). [file 2602322.f1.zip › Supplementary table 1.docx]

| Table S1. Detail of MYOF co-expression genes (TIMER). | | | | | | | |
| --- | --- | --- | --- | --- | --- | --- | --- |
| **Query** | **Statistic** | **P-value** | **FDR (BH)** | **Query** | **Statistic** | **P-value** | **FDR (BH)** |
| **MYOF** | 1.00 | 1.00E-43 | 1.00E-39 | **COQ10A** | -0.74 | 1.15E-32 | 4.74E-30 |
| **AHNAK2** | 0.81 | 3.09E-43 | 2.35E-39 | **QDPR** | -0.74 | 1.28E-32 | 5.16E-30 |
| **PLA2R1** | 0.81 | 3.56E-43 | 2.35E-39 | **C7orf55** | -0.74 | 1.46E-31 | 4.88E-29 |
| **FGD6** | 0.81 | 9.02E-43 | 4.46E-39 | **ABHD14A** | -0.73 | 3.32E-31 | 1.01E-28 |
| **MET** | 0.80 | 9.63E-42 | 3.81E-38 | **GKAP1** | -0.73 | 6.36E-31 | 1.82E-28 |
| **OSBPL3** | 0.80 | 2.05E-41 | 6.75E-38 | **ERP29** | -0.73 | 1.14E-30 | 3.08E-28 |
| **MYO1E** | 0.80 | 2.42E-41 | 6.84E-38 | **NRL** | -0.73 | 1.25E-30 | 3.34E-28 |
| **YAP1** | 0.80 | 3.50E-41 | 8.64E-38 | **PEMT** | -0.73 | 1.98E-30 | 5.07E-28 |
| **PLS3** | 0.80 | 1.00E-40 | 2.20E-37 | **PDXP** | -0.72 | 4.49E-30 | 1.10E-27 |
| **RUNX1** | 0.80 | 1.59E-40 | 3.14E-37 | **CYB5D2** | -0.72 | 9.56E-30 | 2.20E-27 |
| **LPCAT2** | 0.80 | 3.25E-40 | 5.83E-37 | **CIRBP** | -0.71 | 3.97E-28 | 6.89E-26 |
| **TGFA** | 0.79 | 2.77E-39 | 4.56E-36 | **SEC11C** | -0.70 | 5.71E-28 | 9.74E-26 |
| **PTPN12** | 0.79 | 5.27E-39 | 8.02E-36 | **RNF167** | -0.70 | 2.09E-27 | 3.31E-25 |
| **GJB2** | 0.78 | 4.72E-38 | 6.54E-35 | **G6PC3** | -0.70 | 2.26E-27 | 3.52E-25 |
| **ARHGAP42** | 0.78 | 4.96E-38 | 6.54E-35 | **C22orf32** | -0.69 | 5.39E-27 | 7.95E-25 |
| **IL1RAP** | 0.78 | 6.27E-38 | 7.67E-35 | **CACNG2** | -0.69 | 5.87E-27 | 8.60E-25 |
| **EPS8** | 0.78 | 6.60E-38 | 7.67E-35 | **TMEM198** | -0.69 | 8.09E-27 | 1.14E-24 |
| **SEMA3C** | 0.78 | 1.41E-37 | 1.50E-34 | **SERGEF** | -0.69 | 1.24E-26 | 1.69E-24 |
| **ITPRIPL2** | 0.78 | 1.44E-37 | 1.50E-34 | **B3GNT1** | -0.69 | 2.50E-26 | 3.17E-24 |
| **VCL** | 0.78 | 2.04E-37 | 2.01E-34 | **P4HTM** | -0.69 | 4.35E-26 | 5.18E-24 |
| **AHNAK** | 0.78 | 2.38E-37 | 2.24E-34 | **TATDN3** | -0.68 | 8.06E-26 | 9.06E-24 |
| **TMOD3** | 0.78 | 5.62E-37 | 5.05E-34 | **SSR4** | -0.68 | 9.42E-26 | 1.04E-23 |
| **KIAA1217** | 0.77 | 7.70E-37 | 6.62E-34 | **C5orf55** | -0.68 | 1.59E-25 | 1.68E-23 |
| **GPRC5A** | 0.77 | 1.04E-36 | 8.57E-34 | **PRPSAP2** | -0.68 | 2.11E-25 | 2.16E-23 |
| **ZFP36L1** | 0.77 | 1.31E-36 | 1.04E-33 | **ANKRD54** | -0.68 | 2.19E-25 | 2.23E-23 |
| **CASP8** | 0.77 | 2.72E-36 | 2.07E-33 | **MED9** | -0.68 | 3.30E-25 | 3.19E-23 |
| **MMP14** | 0.77 | 4.03E-36 | 2.95E-33 | **C12orf34** | -0.67 | 7.04E-25 | 6.48E-23 |
| **ITGB6** | 0.77 | 5.29E-36 | 3.74E-33 | **TRMU** | -0.67 | 9.37E-25 | 8.46E-23 |
| **ANO1** | 0.77 | 6.72E-36 | 4.58E-33 | **LOC729991** | -0.67 | 9.42E-25 | 8.46E-23 |
| **RALB** | 0.77 | 9.05E-36 | 5.97E-33 | **ATP2A3** | -0.67 | 1.19E-24 | 1.05E-22 |
| **TPM4** | 0.77 | 1.33E-35 | 8.31E-33 | **PELP1** | -0.67 | 1.25E-24 | 1.10E-22 |
| **DOCK5** | 0.77 | 1.34E-35 | 8.31E-33 | **LOC100303728** | -0.67 | 1.28E-24 | 1.12E-22 |
| **TES** | 0.76 | 6.02E-35 | 3.61E-32 | **NANOS1** | -0.67 | 1.42E-24 | 1.22E-22 |
| **ANO6** | 0.76 | 6.84E-35 | 3.98E-32 | **POMT1** | -0.67 | 1.99E-24 | 1.66E-22 |
| **AFAP1** | 0.76 | 8.66E-35 | 4.89E-32 | **MBLAC1** | -0.67 | 2.00E-24 | 1.67E-22 |
| **SERPINB5** | 0.76 | 1.21E-34 | 6.63E-32 | **SAPS2** | -0.66 | 4.53E-24 | 3.59E-22 |
| **MXRA5** | 0.76 | 2.88E-34 | 1.54E-31 | **HDHD2** | -0.66 | 7.40E-24 | 5.66E-22 |
| **OSMR** | 0.76 | 3.95E-34 | 2.05E-31 | **MMAB** | -0.66 | 7.55E-24 | 5.70E-22 |
| **ANXA2P2** | 0.75 | 8.37E-34 | 4.24E-31 | **RPAIN** | -0.66 | 8.25E-24 | 6.16E-22 |
| **INPP4B** | 0.75 | 8.82E-34 | 4.36E-31 | **EML5** | -0.66 | 1.00E-23 | 7.28E-22 |
| **LAMC2** | 0.75 | 1.35E-33 | 6.46E-31 | **SLC26A11** | -0.66 | 1.21E-23 | 8.56E-22 |
| **RYK** | 0.75 | 1.37E-33 | 6.46E-31 | **ATP6V0A1** | -0.66 | 1.83E-23 | 1.26E-21 |
| **FRRS1** | 0.75 | 2.36E-33 | 1.09E-30 | **ATP6V0E2** | -0.66 | 1.89E-23 | 1.29E-21 |
| **FRMD6** | 0.75 | 3.36E-33 | 1.51E-30 | **C3orf71** | -0.66 | 2.22E-23 | 1.50E-21 |
| **PTPN14** | 0.75 | 5.11E-33 | 2.25E-30 | **CARKD** | -0.66 | 3.04E-23 | 2.01E-21 |
| **REEP3** | 0.75 | 8.12E-33 | 3.49E-30 | **CDKN2AIPNL** | -0.65 | 3.78E-23 | 2.46E-21 |
| **ANXA3** | 0.75 | 8.88E-33 | 3.74E-30 | **FBXO9** | -0.65 | 4.79E-23 | 3.07E-21 |
| **TSKU** | 0.74 | 1.46E-32 | 5.77E-30 | **FBLL1** | -0.65 | 5.04E-23 | 3.21E-21 |
| **LAMB3** | 0.74 | 1.49E-32 | 5.77E-30 | **DUSP15** | -0.65 | 6.37E-23 | 3.88E-21 |
| **GJB3** | 0.74 | 7.64E-32 | 2.90E-29 | **ZNF821** | -0.65 | 8.29E-23 | 4.92E-21 |
